# Reduced Enzymatic Browning in Potato Tubers by Specific Editing of a Polyphenol Oxidase Gene *via* Ribonucleoprotein Complexes Delivery of the CRISPR/Cas9 System

**DOI:** 10.3389/fpls.2019.01649

**Published:** 2020-01-09

**Authors:** Matías Nicolás González, Gabriela Alejandra Massa, Mariette Andersson, Helle Turesson, Niklas Olsson, Ann-Sofie Fält, Leonardo Storani, Cecilia Andrea Décima Oneto, Per Hofvander, Sergio Enrique Feingold

**Affiliations:** ^1^Consejo Nacional de Investigaciones Científicas y Técnicas (CONICET), Buenos Aires, Argentina; ^2^Laboratorio de Agrobiotecnología, INTA - EEA Balcarce, Balcarce, Argentina; ^3^Facultad de Ciencias Agrarias, Universidad Nacional de Mar del Plata, Balcarce, Argentina; ^4^Department of Plant Breeding, Swedish University of Agricultural Sciences, Alnarp, Sweden

**Keywords:** CRISPR/Cas9, potato, genome editing, enzymatic browning, polyphenol oxidase, ribonucleoprotein complexes

## Abstract

Polyphenol Oxidases (PPOs) catalyze the conversion of phenolic substrates to quinones, leading to the formation of dark-colored precipitates in fruits and vegetables. This process, known as enzymatic browning, is the cause of undesirable changes in organoleptic properties and the loss of nutritional quality in plant-derived products. In potato (*Solanum tubersoum* L.), PPOs are encoded by a multi-gene family with different expression patterns. Here, we have studied the application of the CRISPR/Cas9 system to induce mutations in the *StPPO2* gene in the tetraploid cultivar Desiree. We hypothesized that the specific editing of this target gene would result in a lower PPO activity in the tuber with the consequent reduction of the enzymatic browning. Ribonucleoprotein complexes (RNPs), formed by two sgRNAs and Cas9 nuclease, were transfected to potato protoplasts. Up to 68% of regenerated plants contained mutations in at least one allele of the target gene, while 24% of edited lines carried mutations in all four alleles. No off-target mutations were identified in other analyzed *StPPO* genes. Mutations induced in the four alleles of *StPPO2* gene, led to lines with a reduction of up to 69% in tuber PPO activity and a reduction of 73% in enzymatic browning, compared to the control. Our results demonstrate that the CRISPR/Cas9 system can be applied to develop potato varieties with reduced enzymatic browning in tubers, by the specific editing of a single member of the *StPPO* gene family.

## Introduction

Polyphenol Oxidases (PPOs; E.C.1.10.3.1, E.C.1.10.3.2, or E.C.1.14.18.1) are copper-containing enzymes, widely distributed among higher plants (Yoruk and Marshall, 2003), that catalyze the oxidation of an extensive range of phenolic compounds to their respective quinones. The quinones generated by action of PPOs can undergo self-polymerization or react with amino acids or free radicals in proteins leading to the formation of dark-colored precipitates (Mayer, 2006). This process, known as Enzymatic Browning, is the cause of reduction in quality that alters the color, taste, texture and nutritional value of several fresh and processed fruits and vegetables (Jukanti, 2017). In addition, the oxidation of polyphenolic compounds by PPOs in plant derived products for human consumption is highly undesirable, since polyphenols are natural antioxidants with possible protective effects against cancer and cardiovascular diseases (Shahidi and Ambigaipalan, 2015).

In potato (*Solanum tuberosum* L.), enzymatic browning is a serious problem for both, producers and the industry, because the tubers can be affected during harvest and post-harvest procedures such as shipping, storage, distribution and blanching (Bachem et al., 1994). This undesired process is controlled in industry by using chemical and/or physical agents (Zhang et al., 2018b). However, these methods have important disadvantages including alterations of organoleptic and nutritional quality of the final products and some of them can even represent a potential risks for human health (Tinello and Lante, 2018). Therefore, the development of new technologies to control PPOs activity in planta is the most promising and safest approach to avoid undesirable browning compounds in fresh and processed potato derived products.

In most of plant species, PPOs are encoded by multi-gene families, which suggests their implication in a variety of cell processes (Tran et al., 2012). PPOs have been associated with several metabolic and biosynthetic processes (Jukanti, 2017) as well as with plant defense responses (Li and Steffens, 2002; Thipyapong et al., 2004; Wang and Constabel, 2004; Kampatsikas et al., 2019). Five *PPO* genes have been originally described in potato (*StPPO*), each one having a special pattern of tissue induction and expression (Thygesen et al., 1995). Once the potato genome sequence data was available (Potato Genome Sequencing Consortium, 2011), a genome-wide survey revealed nine *StPPO*-like genes (named *StPPO1* to *9*), with differential prevalence of ESTs found from different potato tissues (Chi et al., 2014). Several reports have described the use of different RNA silencing technologies to down regulate *StPPO* genes, in order to reduce the enzymatic browning in the tubers (Bachem et al., 1994; Coetzer et al., 2001; Rommens et al., 2006; Llorente et al., 2011). Most of these reports are based on down-regulation of multiple *StPPO* genes, which could have a negative impact on other functions of the enzyme in the plant. Moreover, with this strategy, the gene constructs of the silencing machinery need to be stably inserted into the genome, which represents a drawback considering the time-consuming and costly process of deregulation of a Genetically Modified Organism (GMO) in several countries (Eckerstorfer et al., 2019).

Chi et al. (2014) studied the contribution of each member of the *StPPO* gene family to the total PPO protein activity in the potato tuber. By using artificial micro-RNAs (amiRNAs) authors down-regulated *StPPO* genes individually or in combinations, concluding that four genes are the main responsible for PPO activity in the tuber. *StPPO2* (PGSC0003DMG400018916) gene is the principal contributor to PPO total protein content, with 55% of the total enzyme, followed by *StPPO1* (PGSC0003DMG400029575) with 25–30% and *StPPO4* (PGSC0003DMG400018917) and *StPPO3* (PGSC0003DMG400018914), together with less than 15%.

Genome editing using the CRISPR/Cas9 system is a powerful tool for crop improvement and has been applied to add or modify several traits in many economically important plant species (Arora and Narula, 2017; Baltes et al., 2017; Scheben et al., 2017; Gao, 2018). In its simplest form, the Cas9 nuclease is guided by one or more RNA molecule/s (sgRNA/s) to a specific target site in the host genome to introduce a double stranded break (DSB) in the DNA (Jinek et al., 2012). Following the induction of this DSB, mutations are introduced by the error-prone DNA repair mechanism of Non Homologous End Joining (NHEJ), (Puchta, 2005). When performed in an exon, this can produce a loss of gene function due to frame shifts or deletions of specific fragments of the coding sequence. Cas9 and sgRNAs can be directly delivered to the cell as a Ribonucleoprotein complex (RNPs), (Woo et al., 2015) an approach that avoids foreign DNA insertions in the plant genome. This strategy has been successfully applied to modify genes in several important crops like maize (Svitashev et al., 2016), bread wheat (Liang et al., 2017) and, more recently, potato (Andersson et al., 2018). Considering the current criteria for the determination of the regulatory status of genome edited crops in Argentina and other countries (Whelan and Lema, 2015; Lema, 2019), this approach could result in the development of crop varieties not subjected to the cumbersome GMO regulation process, and treated under the same regulatory framework as varieties obtained by conventional breeding, which includes chemical or radiation mutagenesis (Eckerstorfer et al., 2019).

In this work, we have studied the editing of the *StPPO2* gene in the tetraploid cultivar Desiree, by using the CRISPR/Cas9 system. The reagents for genome editing were delivered in the form of RNPs into potato protoplasts, aiming to avoid the insertion of foreign DNA. Regenerated lines were screened for induced mutations in the target gene and potential off target activity on other members of *StPPO* gene family. Selected lines with mutations in the four alleles of the target gene were grown and assayed for enzymatic browning and PPO activity levels in tubers.

## Results

### SgRNA Design on *StPPO2* Gene and Off Target Prediction

**Figure 1:**
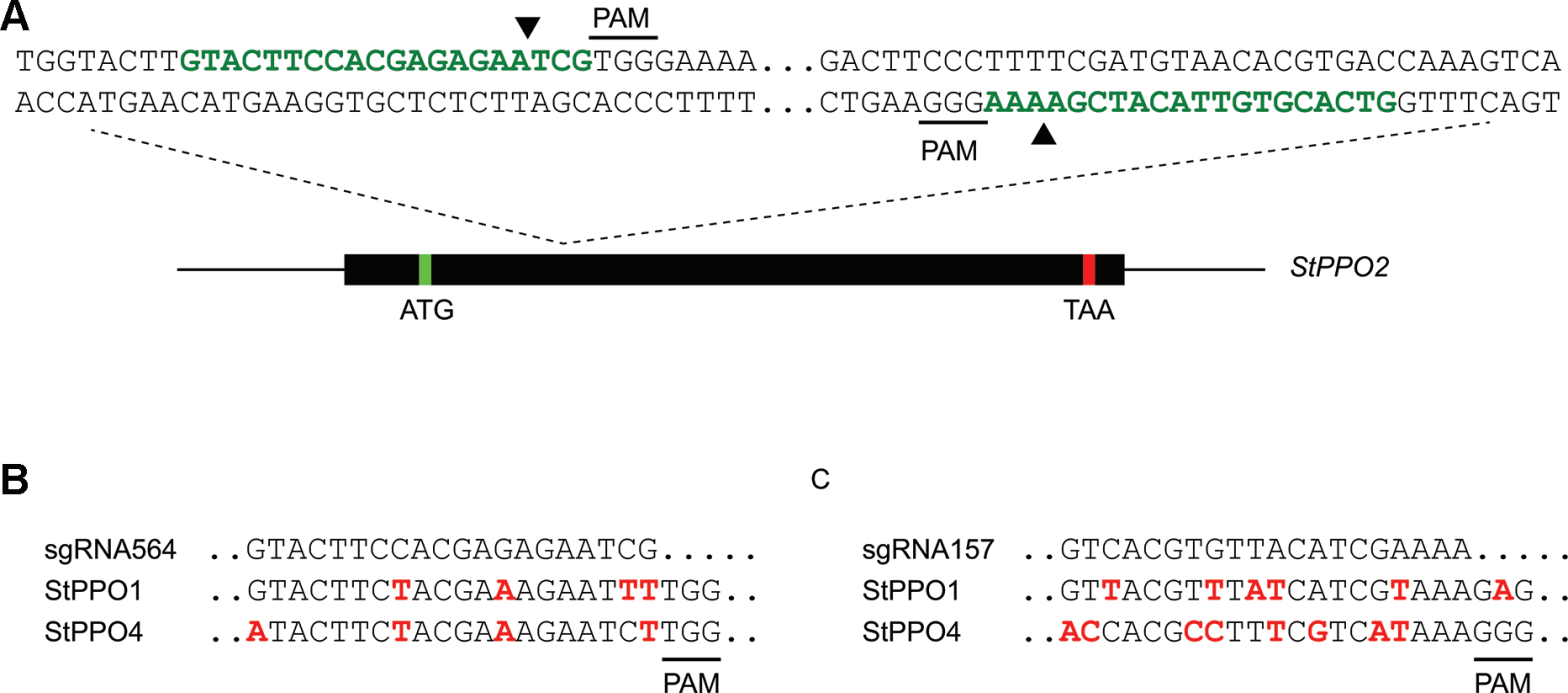
sgRNA design on the *StPPO2* gene and off target prediction **(A)** The structure of *StPPO2* gene is shown with the theoretical positions of the start (ATG, green box) and stop codons (TAA, red box). The partial sequence used for sgRNA design is shown above the gene structure. Targets sites for sgRNAs are marked in green letters and the PAM (5′-NGG-3′) of each target site is indicated. Black arrowheads indicate the predicted cut site for the Cas9 nuclease **(B)** Predicted off target sites for sgRNA564 on *StPPO1* and *StPPO4* genes, with mismatches marked in red letters **(C)** Alignment of sgRNA157 with *StPPO1* and *StPPO4* genes, with mismatches marked in red letters.

In order to find targets to direct Cas9 nuclease to the *StPPO2* gene, a fragment covering the 5´end of the coding sequence was amplified from *S. tuberosum* cv. Desiree and sequenced. The amplified fragment was predicted to encode the N-terminal of the enzyme, including the first copper-binding site (CuA; **Supplementary Figure S1**), which forms part of the active site (Marusek et al., 2006). Two sgRNAs were selected on the resulting sequence with strict absence of allelic variation and named sgRNA157 and sgRNA564 (**Figure 1A**). The expected cutting sites for Cas9 on each target were estimated to be separated by 111 bp on the *StPPO2* sequence (**Supplementary Figure S1**).

In order to avoid inducing mutations in other *StPPO* genes, the two selected sgRNAs were analyzed for possible off target activity. Considering up to four mismatches (Hahn and Nekrasov, 2019) *StPPO1* and *StPPO4* genes were identified as possible off targets of sgRNA564 (**Figure 1B** and **Supplementary Figures S2** and **S3**). Four mismatches at positions +1, +2, +8, and +13 from the Protospacer Adjacent Motif (PAM) were identified in the potential off target site on *StPPO1* and four mismatches at positions +1, +8, +13, and +20, in the potential off target site on *StPPO4* (**Figure 1B**).

No putative off targets on *StPPO* genes were found for sgRNA157 considering four or less mismatches. **Figure 1C** shows the alignment of sgRNA157 with the corresponding sequences of *StPPO1* and *StPPO4*. Although not considered as possible off targets according to the mentioned parameters, both regions were included for further analysis. As highlighted, five mismatches were identified between *StPPO1* and sgRNA157. In addition, a non-canonical PAM sequence (NAG) was found at the 3′ end of the *StPPO1* gene sequence (**Figure 1C**). Eight mismatches were identified between sgRNA157 and the corresponding sequence of the *StPPO4* gene (**Figure 1C**).

### Protoplast Transfection With RNPs and Mutation Screening of Regenerated Lines

**Figure 2:**
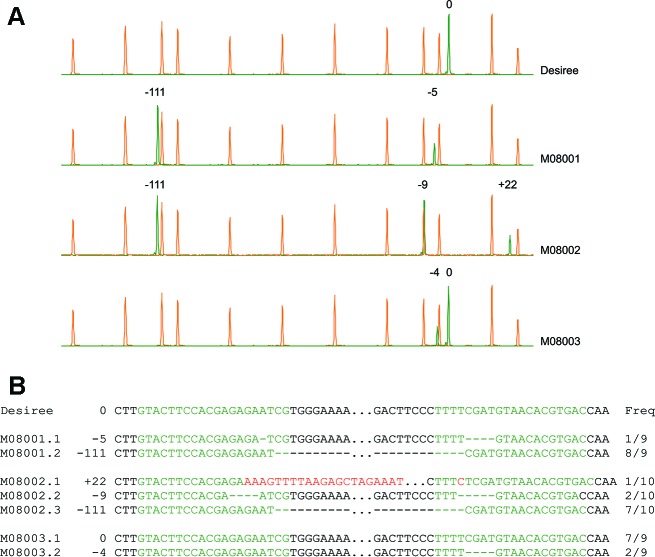
Identification of edited lines using High Resolution Fragment Analysis (HRFA) and characterization of mutations by sequencing **(A)** Electropherograms of HRFA obtained for wild type Desiree and lines M08001, M08002, and M08003. The orange peaks correspond to the elution points of the size standard and green peaks correspond to elution of the *StPPO2* gene fragments. The elution of the wild type fragment is set to 0 and the number of bases inserted (+) or deleted (−) in each fragment is indicated above the respective peak **(B)** Sequencing of a partial fragment of the *StPPO2* alleles in selected lines. Target sites for the sgRNAs are marked in green letters. Deleted nucleotides are indicated as hyphens and inserted bases are marked in red letters. The frequencies obtained during Sanger analysis are indicated, as the number of clones carrying each allelic variant related to the total number of sequenced clones.

CRISPR/Cas9 was delivered in the form of Ribonucleoprotein complexes (RNPs, Andersson et al., 2018) into protoplasts by transfections with 25 or 40% Polyethylenglycol 4000 (PEG) and incubations times of 3 or 30 min, respectively. After regeneration, the identification of edited lines was carried out using the High Resolution Fragment Analysis (HRFA, **Figure 2A**). Based on the analysis of 64 lines regenerated from the 25% PEG transfection, the genome editing efficiency was 27%, defined as the percentage of analyzed lines carrying mutations in at least one allele of the target gene. On the other hand, from the 40% PEG transfection, 28 regenerated lines were analyzed and 68% were found to carry mutations. Taking both transfections together, nine edited lines displayed mutations in all the four alleles of the target gene, with eight of these lines originated from the 40% PEG transfection (**Table 1**). The majority of mutations were small deletions, but in several lines, larger deletions from 102 to 118 nucleotides were observed (**Table 1**), suggesting that Cas9 nuclease introduced cuts at both targets sites, leading to the elimination of the fragment in between. In addition, insertions ranging from 22 to 302 bp were identified in nine lines (**Table 1**). Finally, more than four allelic variants suggesting chimerism was not observed in any of the 92 analyzed lines (**Table 1**).

**Table 1:** Edited lines per experiment detected by HRFA.

Line	Transfection	Allelic Variants
**Desiree RC**	–	0
**M07006**	25% PEG	−4/0
**M07009**	25% PEG	−4/−1/0
**M07014**	25% PEG	−4/0
**M07020**	25% PEG	−102/−4/−1/0
**M07028**	25% PEG	0/+46
**M07029**	25% PEG	−6/0
**M07030**	25% PEG	−5/0
**M07031**	25% PEG	−111/0/+1
**M07032**	25% PEG	−4/0
**M07036**	25% PEG	−5/0
**M07046**	25% PEG	−1/0
**M07051**	25% PEG	0/+1
**M07053**	25% PEG	−6/−4/0/+1
**M07056**	25% PEG	−7/−4
**M07057**	25% PEG	−1/0
**M07062**	25% PEG	−1/0/+1
**M07063**	25% PEG	−3/0/+1
**M07066**	25% PEG	0/+1
**M08001**	40% PEG	−111/−5
**M08002**	40% PEG	−111/−9/+22
**M08003**	40% PEG	−4/0
**M08007**	40% PEG	−9/−5/−3/0
**M08008**	40% PEG	−111/−15/+121/+302
**M08009**	40% PEG	−112/−5/+44
**M08012**	40% PEG	0/+1
**M08013**	40% PEG	−115/−8/−5/+55
**M08014**	40% PEG	−5/−4/0
**M08015**	40% PEG	−111/0/+98
**M08016**	40% PEG	−4/0/+45
**M08017**	40% PEG	−14/−8/0
**M08018**	40% PEG	−114/−11/−5/0
**M08020**	40% PEG	−1/0/+48
**M08024**	40% PEG	−116/−111/−55/+58
**M08025**	40% PEG	−18/−1/0
**M08026**	40% PEG	−4/−2/0
**M08027**	40% PEG	−118/−111
**M08028**	40% PEG	−113/−5/+1

The transfection conditions and the allelic variants found in the analysis of all four alleles of StPPO2 are indicated per line. The number 0 indicates the presence of the wild type allele. Alleles with insertions or deletions are indicated as the number of base pairs with a minus (−) or a plus (+) sign, respectively.

### Sequence Analysis of *StPPO2* in Selected Lines

Sequence analysis was performed on selected lines to confirm HRFA results (**Figure 2B** and **Supplementary Figure S4**). In lines M07056, M08001 and M08002, small deletions were identified, which in most alleles were the product of mutations induced at both target sites, without the elimination of the fragment in between (**Figure 2B** and **Supplementary Figure S4**). In the case of M07056, all mutations are predicted to change the reading frame of the *StPPO2* coding sequence (**Supplementary Figure S4**). The loss of the fragment spanned by the two sgRNAs target sites, was confirmed in alleles of lines M08001, M08002 (**Figure 2B**), and M08008 (**Supplementary Figure S4**), as was indicated by the HRFA results. The presence of the wild type allele was confirmed in line M08003, along with at least one allele carrying a deletion of 4 bp on the target site of sgRNA157 (**Figure 2B**). Moreover, the lower prevalence of the mutated allele in comparison with the wild type in the sequence analysis, suggest that M08003 possess multiple copies of the wild type allele (**Figure 2B**).

Finally, insertions observed in the HRFA were analyzed in lines M08008 and M08002. The larger insertions were found to correspond to fragments of genomic DNA of potato as well as elements of DNA used for the *in vitro* transcription of the sgRNAs (**Figure 2B** and **Supplementary Figure S4**).

### Analysis of Off Target Mutations in *StPPO* Genes

**Figure 3:**
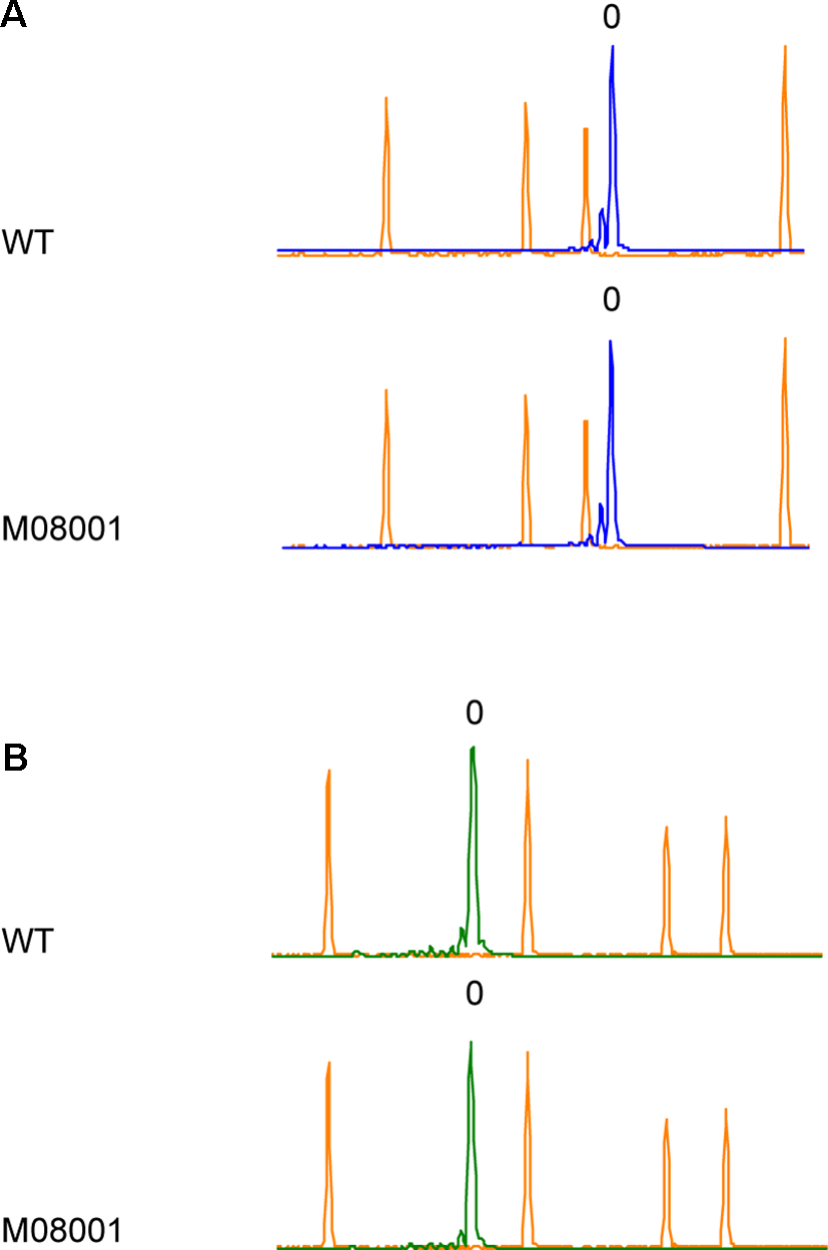
Screening for off target mutations on *StPPO1* and *StPPO4* genes by HRFA. Electropherograms of wild type Desiree and line M08001 are shown. The orange peaks correspond to the elution points of the size standard and the elution of the respective wild type fragment is set to 0 (A) Blue peaks correspond to elution of the *StPPO1* gene fragments (B) Green peaks correspond to elution of the *StPPO4* gene fragments.

Unexpected mutations have been reported in plants using CRISPR/Cas9 as a genome editing tool (Zhang et al., 2018a). With the aim of analyzing the presence of off target mutations on other *StPPO* genes, HRFA was performed on *StPPO1* and *StPPO4* genes in selected lines carrying mutations in all the four alleles of *StPPO2* gene. The electropherograms analysis revealed no differences in fragments length between the edited lines and the control (**Figures 3A**, **B** and **Supplementary Figure S5**), indicating no insertions or deletions introduced on the possible recognition sites for the two sgRNAs.

### Enzymatic Browning and PPO Activity Analysis in Tubers

Selected lines carrying mutations in all four alleles of the *StPPO2* gene were subjected to phenotypic analysis of enzymatic browning and PPO activity in tubers. A wild type line obtained from the regeneration of non-transfected protoplasts were used as a control (Desiree RC). Line M08003 was also included, since it presents a mutation in at least one allele of the target gene, together with at least one copy of the wild type allele (**Figure 2B**). All lines were grown in a growth chamber and displayed no evident phenotypic abnormalities during plant development.

**Figure 4:**
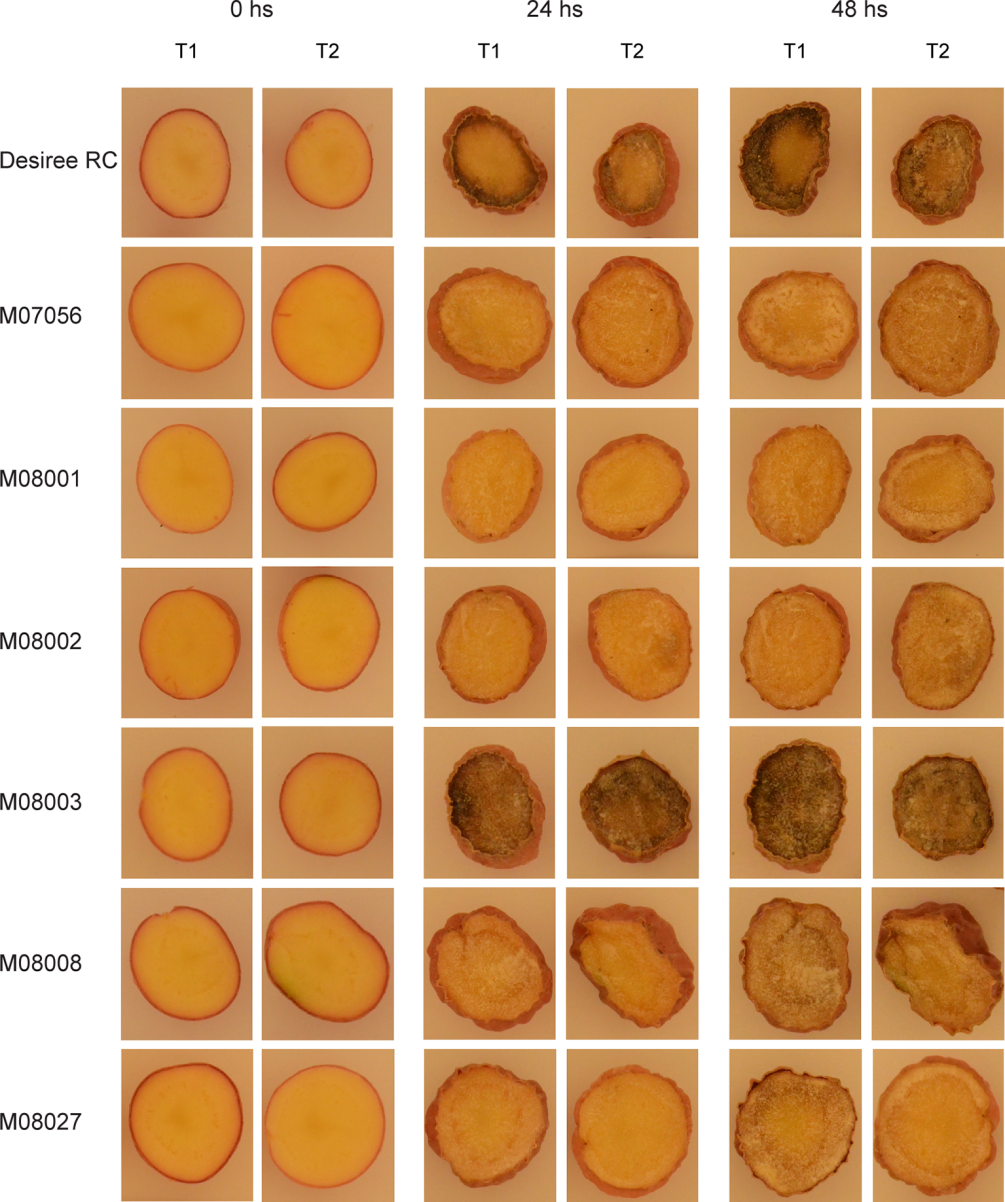
Discoloration development of selected edited lines at times 0, 24, and 48 h after cutting. Two tubers were randomly selected for each edited line and the control, cut and exposed to the air for 48 h at room temperature (24°C). Photos were taken immediately after cutting (0hs), 24 and 48 h later. T1 and T2 indicate Tuber 1 and Tuber 2 of each line, respectively.

For phenotype analysis, the tubers were cut, exposed to air and discoloration development was registered at times 0, 24, and 48 h after cutting (**Figure 4**). After 24 h of air exposure, the typical brown discoloration related to oxidation was visible in lines Desiree RC and M08003, but not in the rest of the analyzed lines (**Figure 4**). The same pattern, but with stronger differences between lines was observed after 48 h of air exposure. Lines Desiree RC and M08003 developed the brown discoloration in a shorter time and over a larger area of the tuber surface (**Figure 4**).

**Figure 5:**
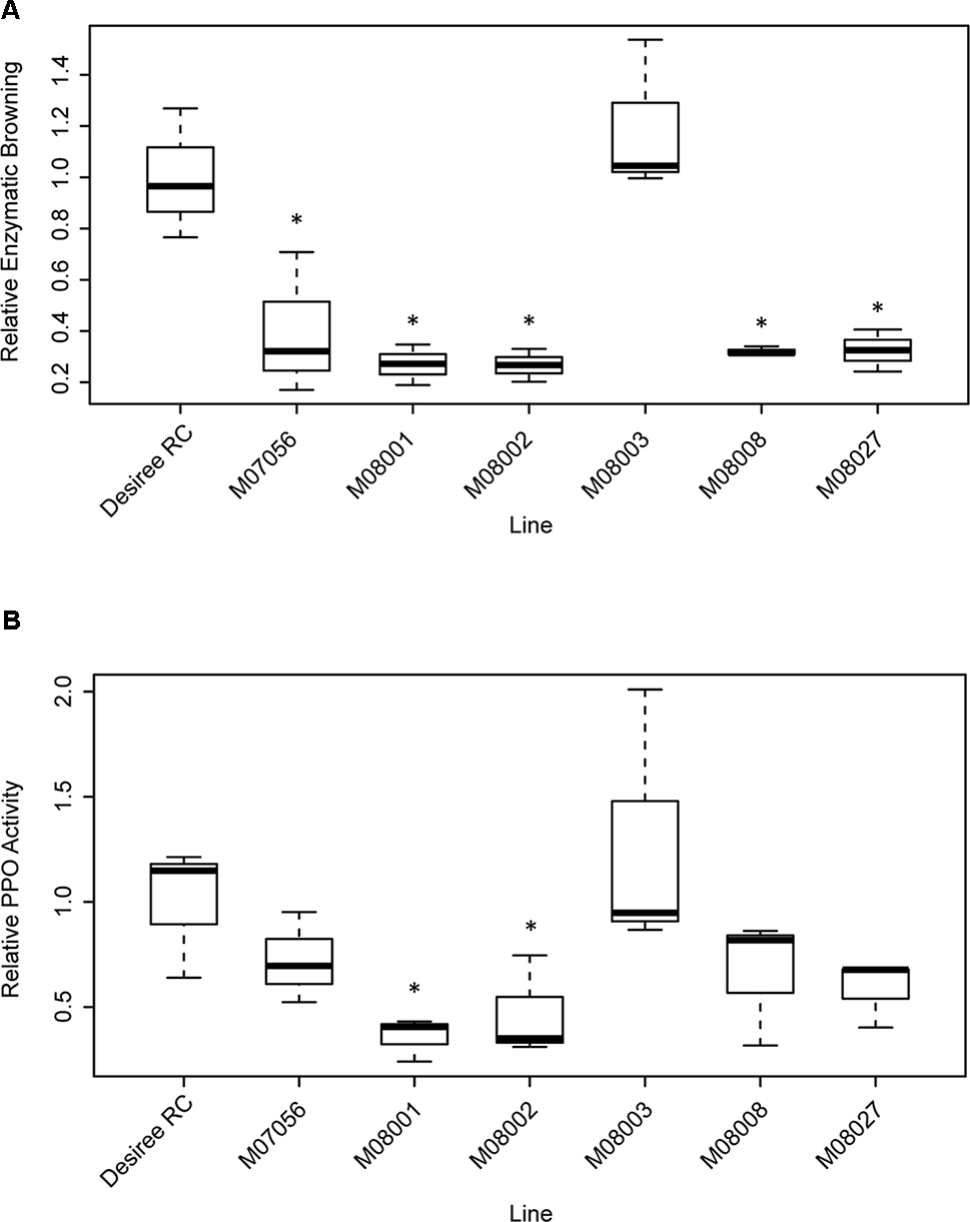
Analysis of Relative Enzymatic Browning **(A)** and Relative PPO Activity **(B)** in tubers of selected edited lines. Each box represents data of three biological replicates of the edited lines and the control Desiree RC, the line across the box represents the median. The box represents the 25^th^ and the 75^th^ percentiles and whiskers represent the maximum and minimum value. Data are relative to the control line Desiree RC. Statistical differences with the control line Desiree RC are denoted *(p < 0.05).

The enzymatic browning was measured for each line and related to that of the control Desiree RC (**Figure 5A**). The relative enzymatic browning was significantly lower in all the edited lines in comparison to the control line (**Table 2**), with the exception of line M08003. The relative enzymatic browning in lines M08001 and M08002 ranged between 0.26 and 0.27, demonstrating a reduction of around 73% related to the control line (**Figure 5A**). Lines M07056, M08008 and M08027, displayed a middle reduction of 68, 67, and 66%, respectively, compared to the control (**Figure 5A**).

**Table 2:** Effect of each line on Relative Enzymatic Browning and Relative PPO Activity variables.

Response variable	Fixed effect	Estimate ± SE	*p*
**Relative Enzymatic Browning**	Intercept	1.00 ± 0.15	<0.001
	M07056	−0.60 ± 0.22	0.01
	M08001	−0.73 ± 0.15	<0.001
	M08002	−0.73 ± 0.15	<0.001
	M08003	0.19 ± 0.22	0.40
	M08008	−0.68 ± 0.14	<0.001
	M08027	−0.67 ± 0.15	<0.001
**Relative PPO Activity**	Intercept	0.48 ± 0.08	<0.001
	M07056	−0.13 ± 0.10	0.2295
	M08001	−0.31 ± 0.09	0.0047
	M08002	−0.25 ± 0.11	0.0355
	M08003	0.13 ± 0.19	0.5137
	M08008	−0.16 ± 0.12	0.2057
	M08027	−0.19 ± 0.09	0.0615

Parameters estimates and p values were taken from Linear Mixed Models with the Line as a fixed effect and the biological replicate as a random effect. p < 0.05 indicates significant differences with the Desiree RC control line.

The PPO activity was measured for each line and made relative to the control Desiree RC (**Figure 5B**). Relative PPO activity was significantly lower in lines M08001 and M08002 in comparison to the control line Desiree RC (**Table 2**) with reductions of 64 and 69%, respectively (**Figure 5B**). Even though not significantly statistical differences, a middle reduction of 39, 28, and 41% in relative PPO activity was observed for lines M07056, M08008, and M08027, respectively, when compared to the control (**Figure 5B**).

In order to determine whether the relative enzymatic browning was correlated to the relative PPO activity in our study, the Spearman correlation coefficient (ρ) was determined between the two variables. As expected, a significantly positive correlation was found (ρ = 0.63, p < 0.005).

## Discussion

Enzymatic browning caused by the activity of PPOs leads to alterations in color and organoleptic properties of fresh and processed fruits and vegetables, which is perceived as a serious quality deficiency for industry and consumers (Yoruk and Marshall, 2003). In our study, the CRISPR/Cas9 system was applied in potato to induce mutations in the *StPPO2* gene, responsible for most of the PPO activity and enzyme content in tubers (Chi et al., 2014). We hypothesized that the specific editing of this target gene would result in a lower PPO activity in the tuber and the consequent reduction of the enzymatic browning.

For the CRISPR/Cas9 system delivery, we utilized Ribonucleoprotein complexes (RNPs) to transfect potato protoplasts and further whole plant regeneration (Nicolia et al., 2015). The genome editing efficiency of 27 and 68% obtained in this study was higher than previously reported using RNPs in potato (9–25%, Andersson et al., 2018). The efficiency in genome editing is largely affected by the target gene as well as the sgRNAs sequence used to direct the Cas9 nuclease (Kumlehn et al., 2018). On the other hand, the activity of CRISPR/Cas9 would be influenced by the transfection efficiency of the reagents into the protoplasts, which could vary between potato varieties. In addition, the combination of two sgRNAs on one target gene used in our study, could explain the increase in the efficiency obtained. Such strategy not only increased the possibilities of inducing mutations in the target gene, but also led to the elimination of larger, specific fragments from the coding sequence as was previously reported in tomato (Brooks et al., 2014), rice (Zhou et al., 2014), barley (Kapusi et al., 2017) and potato (Tuncel et al., 2019; Veillet et al., 2019).

The HRFA performed in our study has shown lines with multiple alleles of *StPPO2* carrying the same type of mutation. Although a less frequent pathway than NHEJ, the DSB repair *via* homologous recombination (HR) is a mechanism observed in plant somatic cells (Puchta, 2005; Shi et al., 2017; Yu et al., 2017). The availability of a mutated homologue allele as a donor template during DSB repair could result in a bias towards homozygous mutations, as observed in the mentioned lines. Nevertheless, our results are not sufficient to confirm such mechanism and further experiments would be necessary to confirm this hypothesis.

Foreign DNA integration into the plant genome is a major concern in genome editing techniques, and is preferably avoided when applied for commercial breeding purposes (Eckerstorfer et al., 2019). This is of special importance in a tetraploid and highly heterozygous crop like potato, since backcrossing techniques to eliminate inserted foreign DNA would lead to the loss of allelic combination in an elite variety (Nadakuduti et al., 2018). In some of the mutated lines identified in our study, insertions were observed in the target region, which corresponded to fragments of the DNA template used in the *in vitro* transcription of the sgRNAs, or potato genomic fragments. Although, the latter cannot be consider as a foreign DNA integration, the first type of insertions could be avoided by using synthetic sgRNA instead, as previously reported by Andersson et al. (2018). Nevertheless, the percentage of insertions detected was very low (9 out of 37 lines) and, in addition, we obtained a majority of multi-allelic edited lines with no evident DNA insertions into the target sites. The confirmation of the absence of foreign DNA in such lines could result in plants considered not different from conventionally bred varieties, taking into account the actual criteria for determining the regulatory status of genome edited products in Argentina and other countries (Whelan and Lema, 2015; Eckerstorfer et al., 2019; Lema, 2019).

Off target activity, i.e. introduction of unintended mutations, have been reported using the CRISPR/Cas9 system in plants (Zhang et al., 2018a). Assaying all possible off target mutations induced by the selected sgRNAs would only be possible throughout whole genome sequencing of the edited lines (Li et al., 2019), a goal that is beyond the objectives of our study. Nevertheless, we aimed to confirm that our selected edited lines displayed mutations only in the *StPPO2* gene, with no alteration in the coding sequences of other members of the *StPPO* gene family, as paralogs may share a considerably degree of sequence similarity (Chi et al., 2014). Only two possible off target sites were found on other *StPPO* genes for sgRNA564, considering up to four mismatches. The HRFA of the selected lines indicated no insertions or deletions in *StPPO1* and *StPPO4* genes. The presence of multiple mismatches into the seed region (defined as the 8–12 nt proximal to the PAM) between the selected sgRNAs and the rest of *StPPO* genes could explain their specificity for *StPPO2* (Hahn and Nekrasov, 2019). On the other hand, the use of RNPs as delivery method for the CRISPR/Cas9 system has been proposed to reduce the incidence of off targets effects, due to the rapid degradation of the Cas9 nuclease and the sgRNAs in the cell (Nadakuduti et al., 2018; Zhang et al., 2018a; Hahn and Nekrasov, 2019).

Earlier studies have reported the use of different RNA silencing technologies to down-regulate the expression of *StPPO* genes in potato tubers (Bachem et al., 1994; Rommens et al., 2006; Llorente et al., 2011). The approach taken in those reports was to reduce the expression of several members of the *StPPO* gene family, which led to a reduction in the enzyme content and enzymatic browning reactions. The contribution of the different members of *StPPO* genes to the total PPO activity was latter established in potato tubers using amiRNA technology (Chi et al., 2014). Despite amiRNAs proved to be efficient in regulating the expression of *StPPO* genes individually or in combination, several off targets effects were observed with lines displaying a moderate to high reduction of non-targeted *StPPO* genes expression (Chi et al., 2014). The reduction in PPO activity was 15–95%, while the reduction in enzymatic browning was 10–65%, depending on the combination of *StPPO* genes down regulated. The greatest reduction, however, occurred when *StPPO1* to *4* were all suppressed. For unknown reasons, the authors could not obtain lines expressing the amiRNA directed to *StPPO2* gene alone. Nevertheless, correlations studies indicated that the expression of *StPPO2* gene was strongly correlated with the levels of PPO activity and enzyme content in tuber. In the present study, we have demonstrated that lines carrying mutations in all the four alleles of *StPPO2* gene displayed a reduction up to 69% and 73% in the PPO activity and enzymatic browning, respectively. Our result not only corroborate the previous report pointing out *StPPO2* as the major contributor to PPO activity in tubers, but also demonstrate that non-browning potatoes can be obtained by the sole induction of mutations in that gene, without affecting other members of the gene family. Our approach could be advantageous in order to avoid the downside effects of reducing the expression of other members of the *StPPO* gene family, affecting their potential involvement in important cell functions (Yoruk and Marshall, 2003; Jukanti, 2017). Furthermore, we have demonstrated that the CRISPR/Cas9 system is a highly efficient tool for inducing mutations in a specific member of a gene family that shares a high identity of nucleotide sequence (Thygesen et al., 1995; Chi et al., 2014).

The phenotypes observed in the selected lines, were correlated with the mutations found in the *StPPO2* gene. Thus, the frame shift mutations (deletions of 4 or 7 bp) in all alleles of the *StPPO2* gene, is the most likely cause for the reduced PPO activity and concurrent reduced enzymatic browning in line M07056. Similar phenotypic effects were observed in lines M08001, M08002, M08008, and M08027. In addition to alleles carrying mutations that produced frame shifts in the coding sequence, alleles with deletions of 111 bp were introduced in *StPPO2* of those lines. Even though this mutation is not expected to produce a frame shift, a large deletion introduced in the coding sequence near the first copper-binding domain, might affect the functionality of the enzyme, if translated. PPOs from a large number of plant species share a conserved structure in the N-terminal domain, which is critical for the function of the enzyme (García-Borrón and Solano, 2002; Marusek et al., 2006; Tran et al., 2012). Similarly, line M08002 presented one allele carrying a deletion of 9 bp, because of a deletion of 4 bp in the target site of sgRNA564 and a deletion of 5 bp in the target site of sgRNA157. Although no frame shifts were detected for the rest of the coding sequence, the frame shift in the region spanning between both target sites may be related to reduction in the enzyme activity, similar to the effect produced by the elimination of such fragment.

It is not established if all the alleles of the *StPPO2* gene contribute equally to the protein activity in the tuber. Based on our sequencing results, line M08003 contained at least one mutated allele of *StPPO2* most likely in combination with multiple copies of wild type allele. This line displayed a PPO activity and enzymatic browning levels almost identical to the control Desiree RC, which indicates that the remaining wild type alleles were sufficient for a normal enzyme function. Previous studies in other plant species have shown allelic variations in *PPO* genes to be associated with differences in the levels of PPO activity (Taketa et al., 2010; Beecher et al., 2012). A more detailed analysis is needed in the case of potato *PPO* genes.

For phenotypic characterization, we performed two analyses on selected edited lines, i.e. Relative Enzymatic Browning and Relative PPO Activity. The two methods produced similar results, with the selected edited lines displaying a reduction in both parameters. In addition, both variables presented a significantly positive correlation between them. Even though a clear reduction in relative PPO activity for lines M07056, M08008, and M08027, no statistical differences were observed relative to the control. This may be due to a higher variance of the values for these lines. Despite the small disparity in the statistical analysis between the variables for the mentioned lines, our results were consistent with all lines displaying a reduced enzyme activity, which turned to a reduced enzymatic browning in the tuber. Lines M08001 and M08002 values were statistically significant with both determinations.

Argentina is one of a few countries to develop legislation to assess regulatory matters regarding genome edited organisms (Whelan and Lema, 2015; Eckerstorfer et al., 2019; Lema, 2019). In this report, we have studied the application of the CRISPR/Cas9 system to produce edited potato plants with a reduced PPO activity and enzymatic browning in tubers. Our system proved to be specific for the target gene, without affecting the coding sequence of other *StPPO* family members and, consequently, their roles in other cell functions. Considering the current criteria for the determination of the regulatory status of genome edited crops in Argentina and other countries, application of this technology could result in plants that do not fall under strict GMOs regulation, which might represent a major advantage in comparison with previous strategies taken for the improvement of the same trait in potato. We consider that our study represents an important step towards the development of potato varieties that maintain the organoleptic, antioxidant and nutritional properties during harvest and post-harvest procedures, without the utilization of potentially harmful browning controlling agents. This advantage results in benefits for the farmer, the potato processing industry, and finally the consumer.

## Materials and Methods

### SgRNA Design on *StPPO2* Gene of *S. tuberosum* cv. Desiree

The available sequences of PGSC0003DMG400018916 (Potato Genome Sequencing Consortium, 2011) and POT32 (GenBank: U22921.1, Thygesen et al., 1995) were aligned and used for primer design, in order to amplify the *StPPO2* gene in *S. tubersoum* cv Desiree (Chi et al., 2014). Primers F_StPPO2 and R_StPPO2 (**Supplementary Table S1**) were used to amplify a fragment from the 5′ end of the target gene, using 10 ng of genomic DNA as a template in a reaction with Phusion High-Fidelity DNA Polymerase (Thermo Fisher Scientific, Waltham, MA, USA). Reaction conditions were 98°C for 1 min, 30 cycles of 98°C 30 s, 60°C 20 s, 72°C 30 s and a final extension of 72°C for 7 min.

PCR products were cloned into the pJET1.2 vector using the CloneJET PCR Cloning Kit (Thermo Fisher Scientific) and transformed to One Shot TOP10 Chemically Competent *E. coli* (Thermo Fisher Scientific), according to manufacturer instructions. Twelve randomly picked colonies were selected for plasmid purification and Sanger sequencing using the primers provided by the CloneJET PCR Cloning Kit. The resulting sequences were aligned to avoid allelic variation during sgRNA design and further High Resolution Fragment Analysis (HRFA) primer design (**Supplementary Figure S1**).

The Cas-Designer Tool^1^ was used for sgRNA design, using one of the sequences obtained for *StPPO2* as a query and *S. tuberosum* (PGSC v4.03) as a target genome (Park et al., 2015). sgRNA157 and sgRNA564 (**Figure 1A**) were selected according to the Out of Frame Score (Bae et al., 2014) and the strict absence of allelic variation along the target sequence (**Supplementary Figure S1**).

### Off Target Sites Prediction on *StPPO* Genes

Cas-OFFinder Tool^2^ was used for possible off targets site identification on other members of the *StPPO* gene family within the genome database of *S. tuberosum* (PGSC v4.03). Searching for sequences with up to 4 mismatches (Hahn and Nekrasov, 2019) with the selected sgRNAs and a 5′-NRG-3′(R = A or G) as PAM sequence, putative off targets were found in the genome at positions 45631511 and 45870133 of the chromosome 8 of potato for sgRNA564. Using the genome browser available on Sol Genomics Network^3^ genes PGSC0003DMG400029575 (*StPPO1*) and PGSC0003DMG400018917 (*StPPO4*) were identified as the only two putative off targets on *StPPO* genes with expression data (Potato Genome Sequencing Consortium, 2011; Chi et al., 2014).

The available sequences of both genes were used for primer design (**Supplementary Table S1**) in order to sequence the putative off targets sites in *S. tuberosum* cv Desiree and confirm the *in silico* analysis. Amplification, cloning and sequencing was performed as previously explained for the target gene. The resulting sequences were aligned (**Supplementary Figures S2** and **S3**) and used for HRFA primer design (**Supplementary Table S1**) for off target analysis. The amplified region for HRFA on each gene included both, the predicted off target sites for sgRNA564 (**Figure 1B**) and the region that aligns with sgRNA157 (**Figure 1C**).

### Ribonucleoprotein Complexes Assembly

The sgRNAs were *in vitro* transcribed (Andersson et al., 2018) using the GeneArt Precision gRNA Synthesis Kit (Thermo Fisher Scientific), according to the manufacturer instructions, with minor modifications. The DNA templates for *in vitro* transcription of sgRNA157 and sgRNA564, were obtained using Fw_IVT157/Rv_IVT157 primers and Fw_IVT564/Rv_IVT564 primers, respectively (**Supplementary Table S1**). After assembly, both DNA templates were purified using the GeneJET PCR Purification Kit (Thermo Fisher Scientific) and quantified using a Trinean DropSense 16 (Techtum, Nacka, Sweden). Thirty ng of DNA were used in each case for *in vitro* transcription for 3 h. After transcription, sgRNAs were treated with 1 unit of DNAse I for 15 min following the instructions of GeneArt Precision gRNA Synthesis Kit, afterwards purified, and quantified using the Trinean DropSense 16.

Right before transfections into potato protoplasts, 5 µg of each sgRNA was mixed with 0.03 nmol of GeneArt Platinum Cas9 Nuclease (Thermo Fisher Scientific) in a final volume of 5 µl and incubated for 15 min at room temperature.

### Protoplasts Transfection and Plant Regeneration

Protoplasts were isolated from 5-week old plantlets according to Nicolia et al. (2015). For transfections, 100,000 protoplasts were incubated with RNPs and 25% Polyethylenglycol (PEG) 4000 (Duchefa Biochemie, Haarlem, The Netherlands) for 3 min, or with RNPs and 40% PEG4000 for 30 min. A regeneration control was included, which consisted of the same number of protoplasts incubated with 40% PEG but no RNPs, for 30 min. After transfections, all protoplasts were embedded in sodium alginate and cultured for calli regeneration, according to Nicolia et al. (2015).

Green calli were released from alginate blobs after 21 days of culture, and subcultured for shoot growth induction. To ensure the analysis of independent lines, one shoot was picked per callus and transferred for root development. Samples from leaves of the full regenerated plantlets were picked for genomic DNA extraction and further analysis.

### Identification of Edited Lines and Sequencing Analysis

Genomic DNA of regenerated plants was extracted from leaves in a 96-Deep well plate. The sampled tissue was homogenized with 500 µl of 100 mM Tris HCl, 50 mM EDTA and 1% SDS, pH 9.0 and 5mm steel beads, using a Retsch Mixer Mill MM400 for 30 s at 30 Hz (Retsch, Haan, Germany). After centrifugation of the tissue debris, DNA was extracted from 200 µl of the cleared lysate, in a QIAcube HT extraction robot using a QIAamp 96 DNA QIAcube HT Kit (QIAGEN, Hilden, Germany) according to the manufacturer instructions.

The presence of mutations in the target gene was determined by High Resolution Fragment Analysis (HRFA), according to Andersson et al. (2017). Primers PPO2_2Bf-HEX and PPO2_2Br (**Supplementary Table S1**) were designed for amplification of the region spanning both sgRNAs target sites on the *StPPO2* gene, taking into account the absence of allelic variation in primers annealing sites in the target gene (**Supplementary Figure S1**). Primers were used to amplify a fragment of 228 bp of the target gene, using Phusion High-Fidelity DNA Polymerase (Thermo Fisher Scientific). Reaction conditions were 98°C for 1 min, 30 cycles of 98°C 30 s, 60°C 20 s, 72°C 15 s, and a final extension of 72°C for 7 min.

Labelled PCR products were analyzed in an Applied Biosystems 3500 Genetic Analyzer (Thermo Fisher Scientific), according to the instructions of manufacturer, using GeneScan 600 LIZ Dye Size Standard (Thermo Fisher Scientific) as internal lane size standard. Fragments length were determined with GeneMarker Software (SoftGenetics, State College, PA, USA) and insertions or deletions were identified comparing each line electropherogram versus the control.

*StPPO2* gene was sequenced by Sanger in selected edited lines to confirm the HRFA results. Primers PPO2_2Bf and PPO2_2Br (**Supplementary Table S1**) were used for PCR amplification of the fragment with the same conditions mentioned above, and the products cloned using the CloneJET PCR Cloning Kit (Thermo Fisher Scientific), as previously. Twelve randomly picked clones were sequenced per line for mutations characterization.

### Off Target Analysis

The presence of putative off target mutations in *StPPO1* and *StPPO4* genes was determined by HRFA as described above. Primers PPO1_OT564_F-6-FAM and PPO1_OT564_R (**Supplementary Table S1**) were used for the analysis of the *StPPO1* gene (**Supplementary Figure S2**). PPO4_OT564_F-HEX and PPO4_OT564_R primers (**Supplementary Table S1**) were used for the analysis of the *StPPO4* gene (**Supplementary Figure S3**).

### Plant Growth Conditions and Tubers Harvesting

Selected *in vitro*-regenerated plantlets were transferred to 1 L pots with substrate and placed in a growth chamber, at a constant temperature of 24°C in a photoperiod of 16 h (120 µmol m^−2^ s^−1^) light–8 h dark. Three biological replicates were grown for each edited line and the control line Desiree RC. Tubers were harvested after 120 days of culture, right before plants senescence.

### Enzymatic Browning and PPO Activity Determinations

Enzymatic Browning and PPO activity were measured according to Chi et al. (2014), with minor modifications. Tubers were randomly selected per each edited line and the control Desiree RC and triple biological replicates were used for the determinations.

For enzymatic browning assay, slices were manually cut from the center of the tubers and immediately frozen in liquid nitrogen. The frozen samples were processed with 5 ml of cold PPO extraction buffer (100 mM sodium phosphate buffer pH 6.0, 2% TX-100, 2% PVPP) using an Ultra-Turrax T-25 (IKA, Königswinter, Germany) at 11,000 rpm for 30 s. Homogenates were allowed to oxidize for 1 h at room temperature, and afterwards aliquots were transferred to 1.5 ml centrifuge tubes and centrifuged for 10 min at 11,000 rpm. The absorbance at 475 nm (A475nm) was measured in 300 µl of the supernatant in a 96 wells plate, using an Epoch Microplate Spectrophotometer (Bioteck, Winooski, VT, USA), with three technical replicates. The total protein concentration of the homogenates were determined using the Pierce BCA Protein Assay Kit (Thermo Fisher Scientific) in the same spectrophotometer and the Enzymatic Browning calculated as the A475nm/mg of total protein. Finally, the Relative Enzymatic Browning was calculated as the value of each line related to the control Desiree RC.

For PPO activity assay, the frozen samples were processed with 5 ml of cold PPO extraction buffer as above, and the homogenates were transferred to 2 ml centrifuge tubes and centrifuged at 11,000 rpm, 4°C during 30 min. The supernatants were transferred to new tubes and kept in ice until PPO activity measurements. PPO activity was measured adding 100 µl of sample into a quartz cuvette and 900 µl of PPO assay buffer (50 mM sodium phosphate buffer pH 6.0, 0.1% SDS and 15 mM 4-Methylcatechol). A SmartSpec3000 Spectrophotometer (Bio-Rad, Hercules, CA, USA) was used to measure the absorbance increase at 400 nm (A400nm) every 5 s for 1 min at 25°C. Three technical replicates were performed for the determinations and one unit (1U) of PPO enzymatic activity was defined as the amount of enzyme necessary to change A400nm in 0.001/min at 25°C. The total protein concentration of each sample was determined using the Pierce BCA Protein Assay Kit (Thermo Fisher Scientific) as before, and enzymatic activity was calculated as U/mg of total protein. Finally, Relative PPO Activity was calculated as the value of each line related to the control Desiree RC.

### Statistical Analysis

Linear Mixed Models were used to test the effect of the different lines in the Relative PPO Activity and Relative Enzymatic Browning variables (Linear Mixed-Effects Models: Basic Concepts and Examples, 2000). We considered each line as a fixed-effect. In order to take in consideration possible variation in the individual plants, we considered the identity of each biological replicate as a random effect. All possible models were evaluated and we compared competitive ones using Akaike Information Criterion (AIC). All the analysis were performed in R^4^ using nlme package.

Spearman's correlation analysis was performed in R, using the measured data of the variables “Relative Enzymatic Browning” and “Relative PPO Activity.”

## Data Availability Statement

All datasets for this study are included in the article/**Supplementary Material**.

## Author Contributions

MG, GM, MA, SF, and PH designed the study. MG, MA, HT, NO, and A-SF planned and conducted the protoplasts transfection, plant regeneration and mutations analysis. MG, GM, LS, and CD planned and conducted the tuber production and phenotype analysis. MG wrote the manuscript, which was revised by GM, MA, SF, and PH. All authors read and approved the final version of the manuscript.

## Funding

This work was funded by INTA PNBIO1131024 “Desarrollo de sistemas alternativos de generación y utilización de variabilidad genética y su aplicación al mejoramiento de los cultivos” and INTA-Fondo de Valorización Tecnológica “Variedades de papa editadas con mayor calidad industrial y nutricional”. In addition, this study was partially financed by Trees and Crops for the Future (TC4F), a Strategic Research Area at SLU, supported by the Swedish Government. MG visit to SLU was possible thanks to a training grant provided by Becar program from the Argentinian Ministry of Education and Sports.

## Conflict of Interest

The authors declare that the research was conducted in the absence of any commercial or financial relationships that could be construed as a potential conflict of interest.

## Footnotes

1 www.rgenome.net/cas-designer

2 www.rgenome.net/cas-offinder

3 www.solgenomics.net

4 www.R-project.org
