# Supplementary material for: Reduced Enzymatic Browning in Potato Tubers by Specific Editing of a Polyphenol Oxidase Gene via Ribonucleoprotein Complexes Delivery of the CRISPR/Cas9 System
Source: Front Plant Sci. 2020 Jan 9;10:1649. doi: 10.3389/fpls.2019.01649 (PMC6962139; doi:10.3389/fpls.2019.01649)
Supplement: Supplementary file 1 [file Table_1.docx]

Supplementary Material

**Figure S1**. Sequence analysis of the *StPPO2* gene in *Solanum tuberosum* cv. Desiree. SNPs are marked as red letters. The coding sequence of the first copper-binding domain (CuA) is highlighted in green and selected sgRNAs, in yellow. The expected cutting site for Cas9 is marked with black arrows and the PAM sequences are underlined.

StPPO2.1 TTTAAGCAACAAGCTAATATACATTGTGCTTATTGTAATGGTGCTTATAGAATTGGTGGC

StPPO2.2 TTTAAGCAACAAGCTAATATACATTGTGCTTATTGTAATGGTGCTTATAGAATTGGTGGC

StPPO2.3 TTTAAGCAACAAGCTAATATACATTGTGCTTATTGTAATGGTGCTTATAGAATTGGTGGC

StPPO2.4 TTTAAGCAACAAGCTAATATACATTGTGCTTATTGTAATGGTGCTTATAGAATTGGTGGC

StPPO2.5 TTTAAGCAACAAGCTAATATACATTGTGCTTATTGTAATGGTGCTTATAGAATTGGTGGC

StPPO2.6 TTTAAGCAACAAGCTAATATACATTGTGCTTATTGTAATGGTGCTTATAGAATTGGTGGC

StPPO2.7 TTTAAGCAACAAGCTAATATACATTGTGCTTATTGTAATGGTGCTTATAGAATTGGTGGC

StPPO2.8 TTTAAGCAACAAGCTAATATACATTGTGCTTATTGTAATGGTGCTTATAGAATTGGTGGC

StPPO2.9 TTTAAGCAACAAGCTAATATACATTGTGCTTATTGTAATGGTGCTTATAGAATTGGTGGC

StPPO2.10 TTTAAGCAACAAGCTAATATACATTGTGCTTATTGTAATGGTGCTTATAGAATTGGTGGC

StPPO2.11 TTTAAGCAACAAGCTAATATACATTGTGCTTATTGTAATGGTGCTTATAGAATTGGTGGC

StPPO2.12 TTTAAGCAACAAGCTAATATACATTGTGCTTATTGTAATGGTGCTTATAGAATTGGTGGC

StPPO2.1 AAAGAGTTACAAGTTCATAATTCATGGCTTTTCTTCCCGTTCCATAGATGGTACTTGTAC

StPPO2.2 AAAGAGTTACAAGTTCATAATTCATGGCTTTTCTTCCCGTTCCATAGATGGTACTTGTAC

StPPO2.3 AAAGAGTTACAAGTTCATAATTCATGGCTTTTCTTCCCGTTCCATAGATGGTACTTGTAC

StPPO2.4 AAAGAGTTACAAGTTCATAATTCATGGCTTTTCTTCCCGTTCCATAGATGGTACTTGTAC

StPPO2.5 AAAGAGTTACAAGTTCATAATTCATGGCTTTTCTTCCCGTTCCATAGATGGTACTTGTAC

StPPO2.6 AAAGAGTTACAAGTTCATAATTCATGGCTTTTCTTCCCGTTCCATAGATGGTACTTGTAC

StPPO2.7 AAAGAGTTACAAGTTCATAATTCATGGCTTTTCTTCCCGTTCCATAGATGGTACTTGTAC

StPPO2.8 AAAGAGTTACAAGTTCATAATTCATGGCTTTTCTTCCCGTTCCATAGATGGTACTTGTAC

StPPO2.9 AAAGAGTTACAAGTTCATAATTCATGGCTTTTCTTCCCGTTCCATAGATGGTACTTGTAC

StPPO2.10 AAAGAGTTACAAGTTCATAATTCTTGGCTTTTCTTCCCGTTCCATAGATGGTACTTGTAC

StPPO2.11 AAAGAGTTACAAGTTCATAATTCTTGGCTTTTCTTCCCGTTCCATAGATGGTACTTGTAC

StPPO2.12 AAAGAGTTACAAGTTCATAATTCTTGGCTTTTCTTCCCGTTCCATAGATGGTACTTGTAC

StPPO2.1 TTCCACGAGAGAATCGTGGGAAAATTCATTGATGATCCAACTTTCGCTTTGCCATATTGG

StPPO2.2 TTCCACGAGAGAATCGTGGGAAAATTCATTGATGATCCAACTTTCGCTTTGCCATATTGG

StPPO2.3 TTCCACGAGAGAATCGTGGGAAAATTCATTGATGATCCAACTTTCGCTTTGCCATATTGG

StPPO2.4 TTCCACGAGAGAATCGTGGGAAAATTCATTGATGATCCAACTTTCGCTTTGCCATATTGG

StPPO2.5 TTCCACGAGAGAATCGTGGGAAAATTCATTGATGATCCAACTTTCGCTTTGCCATATTGG

StPPO2.6 TTCCACGAGAGAATCGTGGGAAAATTCATTGATGATCCAACTTTCGCTTTGCCATATTGG

StPPO2.7 TTCCACGAGAGAATCGTGGGAAAATTCATTGATGATCCAACTTTCGCTTTGCCATATTGG

StPPO2.8 TTCCACGAGAGAATCGTGGGAAAATTCATTGATGATCCAACTTTCGCTTTGCCATATTGG

StPPO2.9 TTCCACGAGAGAATCGTGGGAAAATTCATTGATGATCCAACTTTCGCTTTGCCATATTGG

StPPO2.10 TTCCACGAGAGAATCGTGGGAAAATTCATTGATGATCCAACTTTCGCTTTGCCATATTGG

StPPO2.11 TTCCACGAGAGAATCGTGGGAAAATTCATTGATGATCCAACTTTCGCTTTGCCATATTGG

StPPO2.12 TTCCACGAGAGAATCGTGGGAAAATTCATTGATGATCCAACTTTCGCTTTGCCATATTGG

StPPO2.1 AATTGGGACCATCCAAAAGGTATGCGTTTTCCTGCCATGTATGATCGTGAAGGGACTTCC

StPPO2.2 AATTGGGACCATCCAAAAGGTATGCGTTTTCCTGCCATGTATGATCGTGAAGGGACTTCC

StPPO2.3 AATTGGGACCATCCAAAAGGTATGCGTTTTCCTGCCATGTATGATCGTGAAGGGACTTCC

StPPO2.4 AATTGGGACCATCCAAAAGGTATGCGTTTTCCTGCCATGTATGATCGTGAAGGGACTTCC

StPPO2.5 AATTGGGACCATCCAAAAGGTATGCGTTTTCCTGCCATGTATGATCGTGAAGGGACTTCC

StPPO2.6 AATTGGGACCATCCAAAAGGTATGCGTTTTCCTGCCATGTATGATCGTGAAGGGACTTCC

StPPO2.7 AATTGGGACCATCCAAAAGGTATGCGTTTTCCTGCCATGTATGATCGTGAAGGGACTTCC

StPPO2.8 AATTGGGACCATCCAAAAGGTATGCGTTTTCCTGCCATGTATGATCGTGAAGGGACTTCC

StPPO2.9 AATTGGGACCATCCAAAAGGTATGCGTTTTCCTGCCATGTATGATCGTGAAGGGACTTCC

StPPO2.10 AATTGGGACCATCCAAAGGGTATGCGTTTTCCTGCCATGTATGATCGTGAAGGGACTTCC

StPPO2.11 AATTGGGACCATCCAAAGGGTATGCGTTTTCCTGCCATGTATGATCGTGAAGGGACTTCC

StPPO2.12 AATTGGGACCATCCAAAGGGTATGCGTTTTCCTGCCATGTATGATCGTGAAGGGACTTCC

StPPO2.1 CTTTTCGATGTAACACGTGACCAAAGTCACCGAAATGGAGCAGTAATCGAT

StPPO2.2 CTTTTCGATGTAACACGTGACCAAAGTCACCGAAATGGAGCAGTAATCGAT

StPPO2.3 CTTTTCGATGTAACACGTGACCAAAGTCACCGAAATGGAGCAGTAATCGAT

StPPO2.4 CTTTTCGATGTAACACGTGACCAAAGTCACCGAAATGGAGCAGTAATCGAT

StPPO2.5 CTTTTCGATGTAACACGTGACCAAAGTCACCGAAATGGAGCAGTAATCGAT

StPPO2.6 CTTTTCGATGTAACACGTGACCAAAGTCACCGAAATGGAGCAGTAATCGAT

StPPO2.7 CTTTTCGATGTAACACGTGACCAAAGTCACCGAAATGGAGCAGTAATCGAT

StPPO2.8 CTTTTCGATGTAACACGTGACCAAAGTCACCGAAATGGAGCAGTAATCGAT

StPPO2.9 CTTTTCGATGTAACACGTGACCAAAGTCACCGAAATGGAGCAGTAATCGAT

StPPO2.10 CTTTTCGATGTAACACGTGACCAAAGTCACCGAAATGGAGCAGTAATCGAT

StPPO2.11 CTTTTCGATGTAACACGTGACCAAAGTCACCGAAATGGAGCAGTAATCGAT

StPPO2.12 CTTTTCGATGTAACACGTGACCAAAGTCACCGAAATGGAGCAGTAATCGAT

**Figure S2.** Sequence analysis of the *StPPO1* gene in *Solanum tuberosum* cv. Desiree. SNPs are marked as red letters. Potential off target of sgRNA564 is highlighted in yellow and the sequence aligned with sgRNA157 is highlighted in green. Sites for HRFA primers annealing are underlined.

StPPO1 TCAATTGGCG/TACGAGTCGAATGAGGGAACTTGATAAAGACTCTTTTGACCC/GTCTTGGGTT

StPPO1 TAAACAACAAGCTAATATTCATTGTGCTTATTGTAACGGTGCTTATAAAGTTGGTGGTAAAGAG

StPPO1 TTGCAAGTTCATTTCTCGTGGCTTTTCTTTCCGTTTCATAGATGGTACTTGTACTTCTACGAAA

StPPO1 GAATTTTGGGATCACTTATTAACGATCCAACTTTTGCTTTACCATAC/TTGGAATTGGGATCAT

StPPO1 CCAAAAGGTATGCGTATACCTCCCATGTTTGATCGTGAGGGGTCATCTCTTTACGATGATAAAC

StPPO1 GTAACCAAAACCATCGCAATGGAACTATTATTGATCTT

**Figure S3**. Sequence analysis of the *StPPO4* gene in *Solanum tuberosum* cv. Desiree. SNPs are marked as red letters. Potential off target of sgRNA564 is highlighted in yellow and the sequence aligned with sgRNA157 is highlighted in green. Sites for HRFA primers annealing are underlined.

StPPO4 CTTATTGTAACGGTGCTTATGCTATTGATGGCAAAGTGTTACAAGTTCATAACTCA/GTGGC

StPPO4 TTTTCTTCCCGTTCCATAGATGGTACTTATACTTCTACGAAAGAATCTTGGGATCACTCATC

StPPO4 AATGATCCAACTTTCGCTTTACCATATTGGAATTGGGATCATCCAAAGGGCATGCGTTTCCC

StPPO4 TCA/CCATGTTTGATATTCCAGGGACTGCCCTTTATGACGAAAGGCGTGGTGAACAAATCCA

StPPO4 TAATGGAACCATTATAGATCTCGGTTCTTTTGGGGATCAAGTTCAGACAACTCAA

**Figure S4.** Alignment of sequenced alleles of *StPPO2* in lines M07056, M08002 and M08008 with the wild type (WT) allele. Target sites for sgRNA157 and sgRNA564 are highlighted in yellow with PAM sequence in bold letters. Green letters indicates DNA insertions from the potato genome and red letters indicates DNA insertions from the DNA template used for in vitro transcription. The frequency of each allele is indicated at the end of the sequence as the number of reads/total of obtained sequences.

WT TTGGTGGCAAAGAGTTACAAGTTCATAATTCATGGCTTTTCTTCCCGTTCCATAGATGGT

M07056.1 TTGGTGGCAAAGAGTTACAAGTTCATAATTCTTGGCTTTTCTTCCCGTTCCATAGATGGT

M07056.2 TTGGTGGCAAAGAGTTACAAGTTCATAATTCATGGCTTTTCTTCCCGTTCCATAGATGGT

WT ACTTGTACTTCCACGAGAGAATCG**TGG**GAAAATTCATTGATGATCCAACTTTCGCTTTGC

M07056.1 ACTTGTACTTCCACGAGAGAATCGTGGGAAAATTCATTGATGATCCAACTTTCGCTTTGC

M07056.2 ACTTGTACTTCCACGAG---ATCGTGGGAAAATTCATTGATGATCCAACTTTCGCTTTGC

WT CATATTGGAATTGGGACCATCCAAAAGGTATGCGTTTTCCTGCCATGTATGATCGTGAAG

M07056.1 CATATTGGAATTGGGACCATCCAAAGGGTATGCGTTTTCCTGCCATGTATGATCGTGAAG

M07056.2 CATATTGGAATTGGGACCATCCAAAAGGTATGCGTTTTCCTGCCATGTATGATCGTGAAG

WT GGACTT**CCC**TTTTCGATGTAACACGTGACCAAAGTCACCGAAATGG Freq

M07056.1 GGACTTCCCTTT----TGTAACACGTGACCAAAGTCACCGAAATGG 5/10

M07056.2 GGACTTCCCTTT----TGTAACACGTGACCAAAGTCACCGAAATGG 5/10

WT TTGGTGGCAAAGAGTTACAAGTTCATAATTCATGGCTTTTCTTCCCGTTCCATAGATGGT

M08002.1 TTGGTGGCAAAGAGTTACAAGTTCATAATTCATGGCTTTTCTTCCCGTTCCATAGATGGT M08002.2 TTGGTGGCAAAGAGTTACAAGTTCATAATTCATGGCTTTTCTTCCCGTTCCATAGATGGT

M08002.3 TTGGTGGCAAAGAGTTACAAGTTCATAATTCTTGGCTTTTCTTCCCGTTCCATAGATGGT

WT ACTTGTACTTCCACGAGAGAATCG**TGG**----------------------GAAAATTCATT

M08002.1 ACTTGTACTTCCACGAGAAAAGTTTTAAGAGCTAGAAATAGCATCGTGGGAAAATTCATT

M08002.2 ACTTGTACTTCCACGA----ATCGTGG----------------------GAAAATTCATT

M08002.3 ACTTGTACTTCCACGAGAGAAT--------------------------------------

WT GATGATCCAACTTTCGCTTTGCCATATTGGAATTGGGACCATCCAAAAGGTATGCGTTTT

M08002.1 GATGATCCAACTTTCGCTTTGCCATATTGGAATTGGGACCATCCAAAAGGTATGCGTTTT

M08002.2 GATGATCCAACTTTCGCTTTGCCATATTGGAATTGGGACCATCCAAAAGGTATGCGTTTT

M08002.3 ------------------------------------------------------------

WT CCTGCCATGTATGATCGTGAAGGGACTT**CCC**TTT-TCGATGTAACACGTGACCAAAGTCA

M08002.1 CCTGCCATGTATGATCGTGAAGGGACTTCCCTTTCTCGATGTAACACGTGACCAAAGTCA

M08002.2 CCTGCCATGTATGATCGTGAAGGGACTTCCCTT------TGTAACACGTGACCAAAGTCA

M08002.3 ------------------------------------CGATGTAACACGTGACCAAAGTCA

WT CCGAAATGG Freq

M08002.1 CCGAAATGG 1/10

M08002.2 CCGAAATGG 2/10

M08002.3 CCGAAATGG 7/10

WT TTGGTGGCAAAGAGTTACAAGTTCATAATTCATGGCTTTTCTTCCCGTTCCATAGATGGT

M08008.1 TTGGTGGCAAAGAGTTACAAGTTCATAATTCTTGGCTTTTCTTCCCGTTCCATAGATGGT

M08008.2 TTGGTGGCAAAGAGTTACAAGTTCATAATTCATGGCTTTTCTTCCCGTTCCATAGATGGT

M08008.3 TTGGTGGCAAAGAGTTACAAGTTCATAATTCATGGCTTTTCTTCCCGTTCCATAGATGGT

M08008.4 TTGGTGGCAAAGAGTTACAAGTTCATAATTCATGGCTTTTCTTCCCGTTCCATAGATGGT

WT ACTTGTACTTCCACGAGAGAATCG**TGG**GAAAATTCATTGATGATCCAACTTTCGCTTTGC

M08008.1 ACTTGTACTTCCACGAGAGAATCGTGGGAAAATTCATTGATGATCCAACTTTCGCTTTGC

M08008.2 ACTTGTACTTCCACGAGAG-ATCGTGGGAAAATTCATTGATGATCCAACTTTCGCTTTGC

M08008.3 ACTTGTACTTCCACGAGAGAAT--------------------------------------

M08008.4 ACTTGTACTTCCACGAGAGAA------ACTTGAAAAAGTGGCACCGAGTCGGTGCTTTCT

WT CATATTGGAATTGGGACCATCCAAAAGGTATGCGTTTTCCTGCCATGTATGATCGTGAAG

M08008.1 CATATTGGAATTGGGACCATCCAAAAGGTATGCGTTTTCCTGCCATGTATGATCGTGAAG

M08008.2 CATATTGGAATTGGGACCATCCAAAAGGTATGCGTTTTCCTGCCATGTATGATCGTGAAG

M08008.3 ------------------------------------------------------------

M08008.4 GAAACTTTTCGATGTAACACGTGACTATAGTGAGTGGAAGTACTATAGTGAGTCGTATTA

WT GGACTT**CCC**TTTT-----------------------------------------------

M08008.1 GGACTTCCCTTTTCGTGGGAAAATTCATTGATGATCCAACTTTCGCTTTGCCA-----TA

M08008.2 GGACTTCCCTTTTAAACAAAGTGAACAAAAAAGAGGAAAAAGCAGAATGGTTATTGATTA

M08008.3 ------------------------------------------------------------

M08008.4 ATTT--------------------------------------------------------

WT ------------------------------------------------------------

M08008.1 TTGGAATTGGGAC----CATCCAAAAGGTATGCGTTTTC-CTGCCATGTATGATCGTGAA

M08008.2 TAGAAATTTGAATGCAAAAACTAAAACATATAATTATCCGATACCAAATAAAATACTAAA

M08008.3 ------------------------------------------------------------

M08008.4 ------------------------------------------------------------

WT ------------------------------------------------------------

M08008.1 GGGACTTCCCTTTATCGTGGGAAAATTCATTGATGATCCAACTTTCGCTTTGCCATATTG

M08008.2 GATAAGTCAAATA-----------------------------------------------

M08008.3 ------------------------------------------------------------

M08008.4 ------------------------------------------------------------

WT ------------------------------------------------------------

M08008.1 GAATTGGGACCATCCAAAGGGTATGCGTTTTCCTGCCATGTATGATCGTGAAGGGACTTC

M08008.2 ------------------------------------------------------------

M08008.3 ------------------------------------------------------------

M08008.4 ------------------------------------------------------------

WT ------------------------------------------------------------

M08008.1 CCTTTTGTAACACGTGACTATAGTGAGTCGTATTAATTTCGAAATTAATACGACTCACTA

M08008.2 ------------------------------------------------------------

M08008.3 ------------------------------------------------------------

M08008.4 ------------------------------------------------------------

WT ---------------------------CGATGTAACACGTGACCAAAGTCACCGAAATGG Freq

M08008.1 TAGTCACGTGTTACATCGAAAAGTTTTCGATGTAACACGTGACCAAAGTCACCGAAATGG 1/12

M08008.2 ---------------------------CGATGTAACACGTGACCAAAGTCACCGAAATGG 3/12

M08008.3 ---------------------------CGATGTAACACGTGACCAAAGTCACCGAAATGG 6/12

M08008.4 ---------------------------CGATGTAACACGTGACCAAAGTCACCGAAATGG 2/12

**Figure S5.** Electropherograms of HRFA on putative off targets sites on selected lines and the control Desiree RC. The orange peaks correspond to the elution points of the size standard, blue peaks correspond to elution of the *StPPO1* gene fragments and green peaks correspond to elution of the *StPPO4* gene fragments.


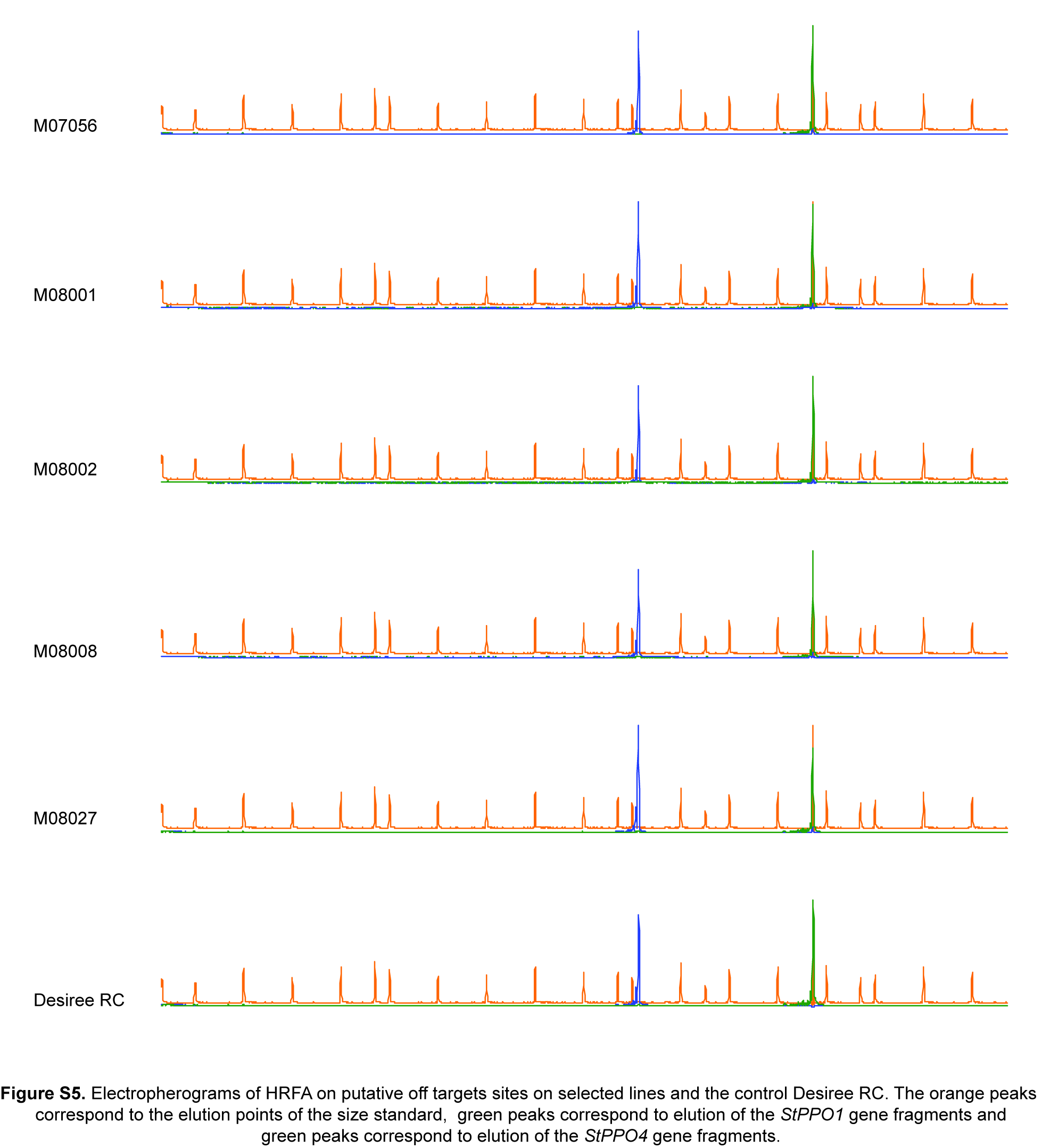


**Table S1**. Primers used in this study.

| Primer ID | Sequence (5´ - 3´) | Purpose |
| --- | --- | --- |
| F_StPPO2 | CTTCCACTCCTAAGCCCTCTTC | Sequence analysis of *StPPO2* gene of *Solanum tubersoum* cv Desiree |
| R_StPPO2 | AGGCCCGCCAAAGAACATCC |  |
| F_StPPO1 | CCTTGACGCTGTTGATAGG | Sequence analysis of *StPPO1* gene of *Solanum tubersoum* cv Desiree for off target validation |
| R_StPPO1 | GACGGACAAGGAGCATTAG |  |
| F_StPPO4 | TCAAGGAAATTGGATTCATTCGTC | Sequence analysis of *StPPO4* gene of *Solanum tubersoum* cv Desiree for off target validation |
| R_StPPO4 | CGCTCCCACGCCTAAAGATA |  |
| Fw_IVT157 | TAATACGACTCACTATAGTCACGTGTTACATCGAAAA | sgRNA DNA template assembly for in vitro transcription of sgRNA157 |
| Rv_IVT157 | TTCTAGCTCTAAAACTTTTCGATGTAACACGTGA |  |
| Fw_IVT564 | TAATACGACTCACTATAGTACTTCCACGAGAGAATCG | sgRNA DNA template assembly for in vitro transcription of sgRNA564 |
| Rv_IVT564 | TTCTAGCTCTAAAACCGATTCTCTCGTGGAAGTA |  |
| PPO2_2Bf-HEX | GCTCCATTTCGGTGACTTT | Amplification of *StPPO2* gene fragment spanning the sgRNAs targets sites for HRFA. PPO2_2Bf were used without labelling for sequence analysis. |
| PPO2_2Br | TGGTGGCAAAGAGTTACAAG |  |
| PPO1_OT564_F-6-FAM | AATAATAGTTCCATTGCGATGGT | Amplification of *StPPO1* gene fragment spanning the possible off target site of sgRNA564 for HRFA |
| PPO1_OT564_R | AAGAGTTGCAAGTTCATTTCTCG |  |
| PPO4_OT564_F-HEX | TGAGTTGTCTGAACTTGATCCC | Amplification of *StPPO4* gene fragment spanning the possible off target site of sgRNA564 for HRFA |
| PPO4_OT564_R | CTTATTGTAACGGTGCTTATGCT |  |
